# Artificial Intelligence and Its Impact on Urological Diseases and Management: A Comprehensive Review of the Literature

**DOI:** 10.3390/jcm10091864

**Published:** 2021-04-26

**Authors:** B. M. Zeeshan Hameed, Aiswarya V. L. S. Dhavileswarapu, Syed Zahid Raza, Hadis Karimi, Harneet Singh Khanuja, Dasharathraj K. Shetty, Sufyan Ibrahim, Milap J. Shah, Nithesh Naik, Rahul Paul, Bhavan Prasad Rai, Bhaskar K. Somani

**Affiliations:** 1 Department of Urology, Kasturba Medical College Manipal, Manipal Academy of Higher Education, Manipal 576104, Karnataka, India; zeeshanhameedbm@gmail.com (B.M.Z.H.); drmilapshah@gmail.com (M.J.S.); bhaskarsomani@yahoo.com (B.K.S.); 2 KMC Innovation Centre, Manipal Academy of Higher Education, Manipal 576104, Karnataka, India; 3 iTRUE (International Training and Research in Uro-Oncology and Endourology) Group, Manipal 576104, Karnataka, India; sufyan.ibrahim2@gmail.com; 4 Curiouz Techlab Private Limited, Manipal Government of Karnataka Bioincubator, Manipal 576104, Karnataka, India; 5 Department of Electronics and Communication, GITAM University, Gandhi Nagar, Rushi Konda, Visakhapatnam 530045, Andhra Pradesh, India; aash.dhavil@gmail.com; 6 Department of Urology, Dr. B.R. Ambedkar Medical College, Bengaluru 560045, Karnataka, India; syed.zahid.raza@gmail.com; 7 Manipal College of Pharmaceutical Sciences, Manipal Academy of Higher Education, Manipal 576104, Karnataka, India; hadiskarimi1997@gmail.com; 8 Department of Information and Communication Technology, Manipal Institute of Technology, Manipal Academy of Higher Education, Manipal 576104, Karnataka, India; hskhanuja2@gmail.com; 9 Department of Humanities and Management, Manipal Institute of Technology, Manipal Academy of Higher Education, Manipal 576104, Karnataka, India; raja.shetty@manipal.edu; 10 Kasturba Medical College Manipal, Manipal Academy of Higher Education, Manipal 576104, Karnataka, India; 11 Department of Mechanical and Manufacturing, Manipal Institute of Technology, Manipal Academy of Higher Education, Manipal 576104, Karnataka, India; 12 Department of Radiation Oncology, Massachusetts General Hospital, Harvard Medical School, Boston, MA 02115, USA; rpaul7@mgh.harvard.edu; 13 Department of Urology, Freeman Hospital, Newcastle NE7 7DN, UK; urobhavan@gmail.com; 14 Department of Urology, University Hospital Southampton NHS Trust, Southampton SO16 6YD, UK

**Keywords:** urology, artificial intelligence, machine learning, urinary incontinence, kidney stone disease, fertility, reproductive urology, renal cell carcinoma, hydronephrosis, urinary reflux, urolithiasis, endourology, pediatric urology, prostate cancer, bladder cancer

## Abstract

Recent advances in artificial intelligence (AI) have certainly had a significant impact on the healthcare industry. In urology, AI has been widely adopted to deal with numerous disorders, irrespective of their severity, extending from conditions such as benign prostate hyperplasia to critical illnesses such as urothelial and prostate cancer. In this article, we aim to discuss how algorithms and techniques of artificial intelligence are equipped in the field of urology to detect, treat, and estimate the outcomes of urological diseases. Furthermore, we explain the advantages that come from using AI over any existing traditional methods.

## 1. Introduction

Advances made in digital technologies, electronic health records, and computing power are producing vast amounts of data in the medical field [1]. With expanded channels, quantity, and quality of data, physicians encounter new obstacles while performing data analysis to establish a reliable diagnosis, planning individualized care, and forecasting the future. Thus, physicians are now relying on artificial intelligence (AI) to build automated models to enhance patient treatment across all aspects of healthcare [2].

In the healthcare industry, AI refers to all the applications, systems, algorithms, and devices that help physicians in providing healthcare based on computer systems and big data. Medical data are ideally used for advising doctors and patients during the decision-making process and identifying the most suitable treatment. The role of AI here is to create new methods for analyzing labor-intensive data, which involves the usage of disciplines of AI. Along with providing improved patient care, it will also enhance efficiency and research and development (R&D), in addition to highlighting disease patterns and correlations earlier than what would be possible via traditional methods. In recent times, AI has seen an explosion in investment and application in the field of medicine, as there is cumulative evidence that it may enhance the delivery of healthcare [3]. This article discusses how AI algorithms and techniques are used in the medical field to detect, treat, and estimate the outcomes of urological diseases and further explains the advantages of using AI over any existing methods.

## 2. Materials and Methods

### 2.1. Search Strategy and Article Selection

A non-systematic review of the literature associated with urology and artificial intelligence that was published between the years 2010 and 2020 was conducted in October 2020 using PubMed and MEDLINE, along with Scopus and Google Scholar. The search strategy involved using a search string based on a set of keywords that included the following: urology, artificial intelligence, machine learning, urinary incontinence, kidney stone disease, fertility, reproductive urology, renal cell carcinoma, hydronephrosis, urinary reflux, urolithiasis, endourology, pediatric urology, prostate cancer, and bladder cancer.

Inclusion criteria:

1. Articles related to artificial intelligence in urology;
2. Original articles of full-text length covering the diagnoses, treatment plans, and results of urologic conditions.

Exclusion criteria:

1. Abstracts, review articles, and chapters from books;
2. Animal, laboratory, or cadaveric studies.

The review of the literature was performed in compliance with the guidelines for inclusion and exclusion criteria. The assessment of titles and abstracts followed by the screening and assessment of the full article text was done according to the inclusion criteria for the selected articles. Further, a manual review of the references list for the chosen articles was conducted to screen for any supplementary work of interest. After a discussion, our authors successfully resolved the disagreements regarding the eligibility for a consensus decision.

### 2.2. What Is Artificial Intelligence?

AI emphasizes constructing an autonomous computer that will effectively execute activities done by humans, using sophisticated non-linear mathematical simulation systems with simple building blocks that replicate human neurons. It begins by searching for ways in which a human mind perceives, understands, and executes cognitive functions. The human mind is capable of intelligence, creativity, language recognition, memory, pattern identification, vision, reasoning, and the creation of ties among facts. AI aims to replicate the aforementioned skills to perform wide-ranging functions, from small, manageable tasks like object recognition to complex tasks like forecasting. AI strategies include learning from known data without bias, dependent only on statistical models, and estimating unknown data about the future, thereby making the task of decision-making smarter and easier [2].

**Figure 1:**
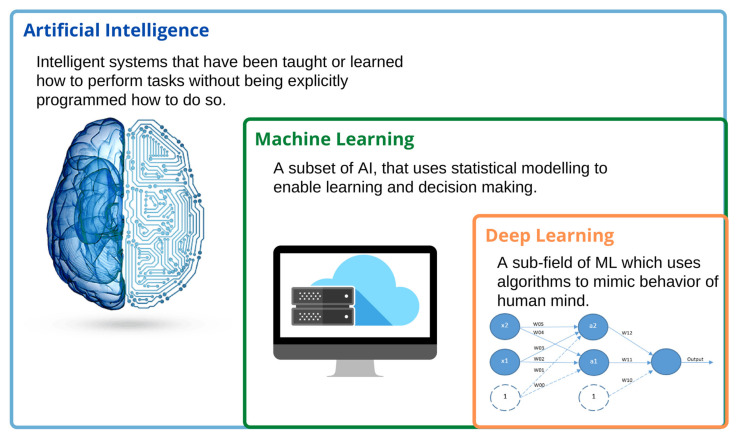
The relationship between artificial intelligence (AI), machine learning (ML), and deep learning (DL).

The ultimate goal of AI is to build a machine that can perceive its environment and perform tasks to maximize its probability of success. The process of achieving this goal is quite complex and involves various AI subfields such as machine learning (ML), artificial neural networks (ANNs) and deep learning (DL), natural language processing (NLP), computer vision, predictive analytics, evolutionary and genetic computing, expert systems, vision recognition, and speech processing, of which most are used in medicine and healthcare today. Thus, some of them need defining for further discussion on the clinical impact of artificial intelligence on various sub-specialties of urology. Figure 1 shows the relationship between artificial intelligence (AI), machine learning (ML), and deep learning (DL).

Machine learning is the process of teaching a computer to make accurate predictions with the help of algorithms that are trained and made to learn from past experiences in a model that maps features to the corresponding outcome variables. The primary aim of ML is to allow the computer to automatically learn when data are fed. An artificial neural network is the basis of deep learning and a subfield of machine learning. ANNs are defined as highly structured information processing units that, along with their synaptic strengths, called weights, mimic the computational abilities of the human brain and nervous system. The neurons are arranged in a series of layers where the weights are modified gradually during the learning process to yield minimum to no error in the input–output mapping. A neural network that has a significant number of layers is called a deep learning network. Being a subfield that holds paramount importance in AI, neural networks have naturally found promising applications in medicine and healthcare, including cardiology, electromyography, electroencephalography, therapeutic drug monitoring for patient care, and sleep apnea.

Decision trees are one of the predictive modeling approaches used in ML, constructed in an algorithmic approach to identifying ways of splitting the dataset based on different conditions. A simple way to describe a decision tree’s working would be to assume a decision node with two or more possible choices. A random forest is an algorithm built with a large number of decision trees that operate as an ensemble. These algorithms are widely adopted in the healthcare industry to determine the patient’s most favorable choice, such as telehealth services.

Another AI subfield that plays a critical role in healthcare is natural language processing, which is concerned with the interaction between the computer and human languages. The biggest challenge in clinical research is to deal with data that are lacking in volume or detail, which is a result of data previously being recorded in narrative clinical documentation. Some of AI’s most promising uses in healthcare include predictive analytics, precision medicine, diagnostic imaging of diseases, and clinical decision support.

### 2.3. Applications of AI in Urology

**Figure 2:**
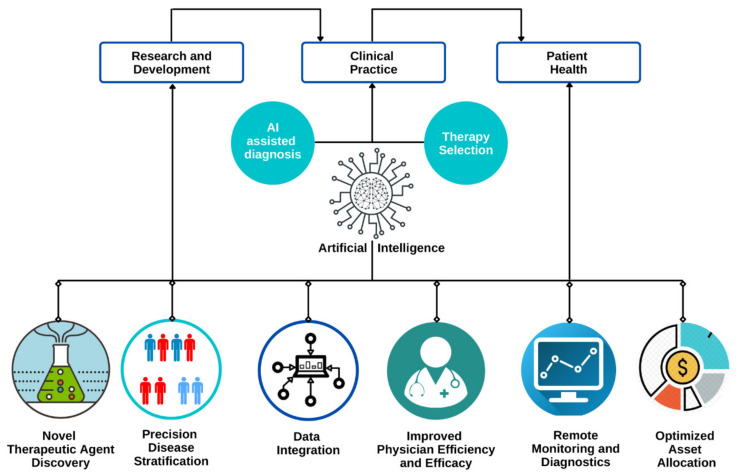
Role of artificial intelligence in urology.

Urology is a field that rapidly expanded through the history of medicine and is continually growing by adopting newer technology to achieve better patient outcomes [4]. Urology being a healthcare segment that deals mainly with male and female urinary tracts and male reproductive organs, the underlying diseases and conditions in these specific areas could become severe if not addressed earlier. Figure 2 shows the role of artificial intelligence in urology.

**Figure 3:**
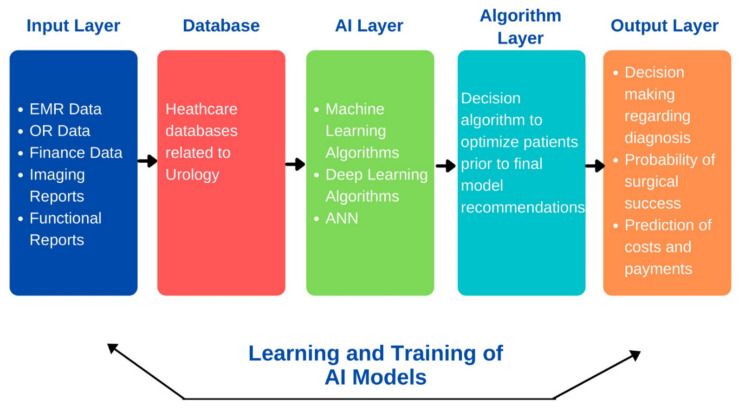
Artificial intelligence in decision making in patients with urological disorders.

AI has been widely adopted in the field for early diagnosis, for providing an effective treatment plan, and in surgical specialties. AI is playing an important role and helping physicians in decision making for patients with urological disorders (Figure 3). In the past 5 years, there has been an emergence of studies affirming the safe and effective augmented-reality (AR) experiences in urology. Modern urologists are using a robotic arm with seven degrees of freedom to remove the kidney remotely, using augmented reality with image overlay [5]. AR is significantly improving the integration of information into the surgical workflow, making minimally invasive procedures less complicated for surgeons. It is bringing innovative approaches in medical education as well as surgical interventions, aiding richer and more interactive experiences. Similarly, there are other technologies combined with AI that impact the field to a great extent. Within urology, there are several sub-specialties, among which urologic oncology, reproductive urology, renal transplant, and pediatric urology are some specialties that have leveraged AI to provide better patient care through developments in diagnostics, treatment planning, and surgical skill assessment [5]. The application of AI in these subfields is discussed below.

## 3. Diagnosis

### 3.1. Urologic Oncology

It is a sub-specialty of urology that is associated with the diagnosis and treatment of cancers in the urinary tract of the human body and male reproductive organs. Urological cancers are relatively common, with prostate, bladder, and kidney cancers among the 10 most prevalent cancers diagnosed in the United States.

### 3.2. Prostate Cancer

The data that are widely used for developing AI algorithms are clinicopathological data of patients abstracted from their electronic medical records (EMRs) because of their high evaluability. With clinical data from 944 Korean patients for predicting organ-confined prostate cancer and non-organ-confined disease, Kim et al. [6] developed a set of ML applications (Table 1). In comparison, Partin tables achieved an accuracy of 66% when using the same dataset. This study highlighted that one can achieve better forecasting results using ML algorithms than using standard statistical models.

Researchers have suggested methods of using AI to simplify the diagnosis and classification of prostate cancer, which has become possible due to the advances in medical imaging and the evidence surrounding it. Using various radiomic features from multiparametric MRI (Magnetic resonance imaging), AI applications have been equipped for detecting prostate cancer [7,8] or for estimating multiparametric MRI Gleason scores [9,10] (Table 1). What also makes AI better than traditional diagnostic standards is its ability to get trained by and learn from complex, multi-variable, big data, thereby improving over time. The ML models displayed an average performance increase of 33–80% for MRI-negative biopsy-positive and 30–60% for MRI-positive biopsy-negative patients when developed using Prostate Imaging Reporting and Data Systems. Fehr et al. [10] observed that ML algorithms had an advantage over unimodal classifiers as they performed more effectively in both identifying the disease and forecasting the correct Gleason score.

Prostate cancer diagnosis depends on the pathologists reviewing specimen slides as well as assessing the same using Gleason scoring, and while the entire procedure takes a lot of time, it can cause intra-observer bias, depending on the experience of the pathologists. AI-assisted image analysis in clinical pathology combines automated image recognition, examination, as well as evaluation of digitalized tissue specimen images, allowing automatic and standardized pathology diagnosis (Table 1). Kwak et al. [11] developed an AI application for detecting the disease in optical pathology images of varying resolutions. The algorithm was able to achieve an accuracy of >97% on the same using segmented prostate specimen images. The aforementioned group also developed ANNs with the nuclear morphology of prostatic epithelial cells for the detection of cancer [12]. They were able to achieve an AUC (Area under the ROC Curve) score of 0.97 for the diagnosis of prostate cancer, surpassing diagnostic methods using handcrafted nuclear engineering technologies. Nguyen et al. [13] developed an ML algorithm to classify the Gleason score of prostate cancer. The classifier has different AUC scores when considering cancer and non-cancer specimens in distinguishing between epithelial tissue and stromal tissue, specifically 0.97 for the former and 0.87 for the latter. In addition, when provided five characteristics of histology, the algorithm achieved an AUC of 0.82 in distinguishing Gleason 3 vs. 4 cancer [13].

### 3.3. Urothelial Cancer

Bladder cancers, also known as urothelial carcinomas, begin in the cell lining of the bladder (i.e., non-muscle-invasive bladder cancer) and can spread to the muscle wall and beyond, to other tissues (i.e., muscle invasive or metastatic bladder cancer). They are highly curable when detected and treated early. Similar to prostate cancer, radiomic imaging and urinary metabolite markers have been used to diagnose urothelial cancer using AI techniques (Table 2). Xu et al. [14] developed ML algorithms with radiomic mpMRI characteristics for distinguishing between bladder tumor and normal bladder wall. Garapati et al. [15] used morphological and textural features of CT (Computed Tomography) urography for determining the stage of bladder cancer. The algorithm was successful in achieving an AUC of 0.7–0.9 in predicting the stage of cancer when using these radiomic attributes. Shao et al. [16] trained decision trees based on urinary metabolic markers to diagnose bladder cancer. They were able to achieve an accuracy of 76.6%, a sensitivity of 71.8%, and a precision of 86.6%. Ikeda et al. [17] used the technique of transfer learning, which enables anomaly detection by using gastroscopic images, to extract important features that apply to cystoscopic images. The dataset used contained 22 cystoscopic images, and the model was compared to results from actual urologists and medical students, who were divided into groups based on their expertise levels. The median time taken by the AI was 5 s as compared to 634 s by the group of observers and achieved 0.930 as the maximum score for Youden’s index.

### 3.4. Renal Cancer

Detection of renal cell cancer (RCC) in its early stages is crucial for its effective treatment, which can be clinically difficult once it spreads. Clinicians can use metabolomics data along with Raman spectra for building AI models, which are effective in the diagnosis of RCC during or before surgery (Table 3). Zheng et al. [18] attempted to identify RCC using a cluster of nuclear-magnetic-resonance-based serum metabolite biomarkers. The authors started with using ANNs to a group and categorized serum metabolites as healthy or RCC and then estimated the detection of RCC in patients individually. Furthermore, ANNs were used for testing patients with RCC who had undergone nephrectomy. The expectation was that an individual patient who was previously classified as RCC would now be healthy after going through a nephrectomy. Haifler et al. [19] used shortwave Raman spectroscopy for distinguishing intra-operatively between healthy and malignant renal tissue. Training an AI model using Raman spectra from RCC and standard tissue samples could improve the identification of benign versus malignant tissue during surgery; the identification currently relies on a frozen section of the pathological specimen [19].

### 3.5. Hydronephrosis/Urinary Reflux

Radiomic imaging technologies are used along with AI to diagnose clinically relevant hydronephrosis and/or urinary reflex. Blum et al. [20] used ML techniques to create a model that is capable of detecting hydronephrosis based on renogram features. The analysis successfully displayed a higher precision in detecting hydronephrosis when compared with just half-time and 30 min clearance. Cerrolaza et al. [21] used ultrasound features to develop ML methods that help in predicting renal obstruction (halftime > 30 min). Logvinenko et al. [22] used ultrasonography results to estimate vesicoureteric reflux (VUR) on the emptying after the cystourethrogram. They found that the AI model worked marginally better than the multivariate logistic regression.

### 3.6. Reproductive Urology

Statistics reveal that around 70 million couples globally are failing to conceive, and male infertility is held responsible for 50% of these cases. Various factors contribute to reproductive problems in men, such as genetic mutations, lifestyle choices, and medical illnesses. Considering such factors, many investigators have paired predictive analytics with AI techniques in their studies to demonstrate how AI could be of assistance in reproductive urology. In the studies by Gil et al. [23] and Candemir et al. [24], AI networks and algorithmic models were used to predict semen quality by considering variables such as lifestyle and environmental factors. Both studies displayed high accuracies, the first study showing an accuracy of ~86% for sperm concentration and 73–76% for motility and the second showing an accuracy of ~90%. These predictive models for semen quality could certainly be used as a tool for screening men with fertility issues to effectively expose any underlying seminal disorders. Among the men, 10–20% undergoing infertility evaluation are found to be suffering from azoospermia, a medical condition in men that causes impotency due to inadequate or no sperm production [25]. Akinsal et al. performed a retrospective study to predict the subset of azoospermic patients that should undergo additional genetic evaluation by applying logistic regression analyses and ANNs [26]. The model identified azoospermic patients with chromosomal abnormalities and those without chromosomal abnormalities with an accuracy of 95%. Exploiting AI to identify individuals with potential genetic abnormalities may mitigate the expense and time lag of formal genetic testing. Apart from predicting semen quality, AI has also been applied in various investigations to determine potential biomarkers for infecundity. In a study by Vickram et al., three different models of ANNs were employed to predict the biochemical parameters for male infertility, of which the backpropagation neural network (BNN) showed minimum error [27]. Men with infertility issues are asked to undergo semen analysis in which most of the parameters, such as sperm motility and concentration, are measured manually. To avoid these time-consuming procedures and the available expensive alternate procedures, Thirumalarjaju et al. introduced an AI-based approach using ANNs that was successful in producing the desired results in analyzing sperm morphology. The network identified abnormal semen samples with a staggering accuracy of 100% [28].

### 3.7. Urolithiasis

There has been a drastic alteration in the way urolithiasis cases are handled now compared with how they were handled in the past, and this approach will be highly influenced by AI techniques [29]. The future of AI in this field could provide complete management for urolithiasis: prevention, diagnosis, and treatment. Kazemi et al. [30] introduced a novel decision support system based on ensemble learning for the early detection (prevention) of kidney stones and explained the underlying mechanisms to determine the type of kidney stones. Various AI algorithms such as the Bayesian model, decision trees, ANNs, and rule-based classifiers were used in this system to understand the complex biological features involved in predicting kidney stones, with the system yielding an accuracy of 97.1%. Längkvist et al. [31] built a CNN (convolutional neural network) model for the detection of ureteral stones in high-resolution CT scans. This model was able to classify stones with a specificity of 100%, where the false positive was found to be 2.68 per scan and the AUC–ROC (receiver operating characteristic curve) was 0.9971.

### 3.8. Pediatric Urology

Pediatric urology handles congenital birth disabilities and disorders in newborn and young children. Though AI is yet to be wholly accepted and explored in this field, it certainly has brought new possibilities to light. About 1–3% of infants suffer from VUR, a condition that could potentially affect the bladder and kidneys if not diagnosed and treated earlier. One of the initial applications of AI in pediatric urology was the use of ANN architectures for the prognosis of VUR. To avoid a painful procedure for VUR detection, such as voiding cystourethrogram (VCUG), that exposes children to radiation, Papadopoulos et al. proposed an ML framework called Venn prediction for detecting VUR [32]. The model exhibited better sensitivity compared with other techniques. Likewise, another novel ML model was suggested to predict the future risk of febrile urinary tract infections (UTIs) related to VUR [33]. The predictive model performed with a reasonable degree of certainty in recognizing children most likely to benefit from VCUG, thus enabling personalized treatment.

### 3.9. Endourological Procedures

Endourology is another area in urology where AI is used to reach novel directions in planning and surgical interventions. Some of the previously mentioned minimally invasive procedures also come under this subfield. Images captured during cystoscopy play a pivotal role in the identification of bladder diseases. Ikeda et al. [17] introduced a support system based on CNNs for the proper diagnosis of bladder cancer using 2102 cystoscopic images. The built model separated the images of normal tissue from those of tumor lesions with high accuracy (area under ROC: 0.98; maximum Youden index (YI): 0.837; sensitivity: 89.7%; and specificity: 94%).

## 4. Outcomes Prediction

Patient outcome predictive analysis requires developing statistical methods that can interpret data to forecast outcomes for a particular patient. We can use either statistical modeling techniques or new methods emerging in the field of AI. These methods have the potential to handle the lack of accuracy and complexity that is typical in clinical and biological data. Additionally, AI techniques can handle the analysis of big data that are too big or too complex for standard statistical models more efficiently [34].

### 4.1. Prostate Cancer

Clinicopathological characteristics of individual patients are used to develop AI algorithms to forecast the outcome. Wong et al. [35] used clinicopathological characteristics of each patient to develop ML algorithms that can estimate the biochemical recurrence following prostatectomy (Table 4). They developed three different ML algorithms that were trained on a dataset of 338 patients to achieve an accuracy between 95% and 98% and an AUC between 0.9 and 0.94. In comparison to the conventional Cox regression analysis, these methods had better predictive efficiency. Tissue morphometric data [36], imaging radiomic features [37,38], and tissue genomic profiling [39,40] are also among the methods that are used for outcome forecasting of a patient. These studies have successfully demonstrated that AI has a higher accuracy when it comes to outcome prediction than other already existing methods.

Apart from the medical causes, surgical performance can also affect patient outcomes. Hung et al. [41,42] created and tested AI algorithms to find out the duration that a patient will have to stay in the hospital and the recovery of urinary control following robotic radical prostatectomy (Table 4). The algorithms were able to achieve an accuracy of 87.2% in the estimation of hospital stay and a C-index of 0.6 for estimating urinary control.

### 4.2. Urothelial Cancer

Urothelial cancers have a high chance of recurrence. AI systems for forecasting cancer recurrence and patient survival have been engineered [43,44,45,46] (Table 5). Lam et al. [43] and Wang et al. [44] used clinicopathological evidence to create and test a significant number of AI algorithms to estimate the 5-year survival after radical cystectomy. Their work results obtained are equivalent to those obtained by other statistical methods. Sapre et al. [45] proposed using an ML classifier with urinary microRNA to diagnose bladder cancer in patients. The classification results by this research achieved an AUC between 0.8 and 0.9 in observing a clinically relevant disease, while also reducing the requirement for cystoscopy by 30%. Bartsch et al. [46] used gene expression profiling to develop AI strategies to forecast the recurrence of non-muscle-invasive bladder cancer. Such experiments have demonstrated the possibility of the potential uses of AI for the treatment of urothelial carcinoma.

### 4.3. Urolithiasis

Percutaneous nephrolithotomy (PCNL) and shockwave lithotripsy (SWL) are commonly recognized therapeutic methods for urolithiasis; however, the rates of success may differ significantly and might include repeat procedures in case the treatment is unsuccessful. Aminsharifi et al. [47] used ANNs to forecast a stone-free PCNL rate with an accuracy of 82.8% and the need to repeat PCNL with an accuracy of 97.7%. Mannil et al. [48] focused their study on the individual patient, using the patient’s body mass index (BMI), along with the 3D texture and scale of the stone, also accounting for the skin-to-stone distance to estimate the performance of SWL. The authors developed and tested five AI algorithms, each with varying 3D textural permutations of patient characteristics, to register AUC values between 0.79 and 0.85, which was an increment from the AUC score of 0.58 that was achieved when using only patient characteristics. For a different report, 3D texture analysis was used to estimate the number of shock waves needed for effective SWL [49]. Against other statistical models, AI displays the most accurate predictions of the number of shock waves needed (<72 or ≥72), with an AUC of 0.838 recorded. Both of Mannil et al.’s [48,49] experiments demonstrated that using AI along with advanced textural analysis methods is practical, reproducible, and predictive of SWL performance.

### 4.4. Renal Transplant

With renal transplantation (RT) being the best available therapy for end-stage renal failure (ESRF), some hindrances are faced in the procedure that can be dealt with by analyzing the survival of transplant patients. The availability of medical data and improving AI techniques have made this challenging prospect more achievable.

The current trend of AI in RT revolves around ensemble learning, where multiple models are combined to achieve better predictive performance. Ethan et al. [50] proposed an ensemble model of ML algorithms for the effective allocation of kidneys by using 18 different predictive variables. The survival model exhibited a higher index of concordance (0.724) than the other existing models (0.68) used for determining recipient priority in the allocation system. Recently, a risk prediction score named iBox has been developed by an international team of French researchers for forecasting the risk of allograft failure after RT [51]. This robust system outperforms the current golden standard (estimated glomerular filtration rate and proteinuria) to monitor kidney recipients. The forecasts of this method, validated on more than 7500 patients, are extremely accurate in decision making, independent of the healthcare environment, medical conditions, clinical action, or actual patient treatment.

Though RT is a better option over dialysis, the recipient’s kidney is always at a risk of rejection, and hence early identification of such complications is necessary. Abdeltawab et al. [52] came up with a non-invasive method for the timely diagnosis of acute RT rejection. The authors developed a novel deep-learning-based computer-aided diagnostic system drawn upon both imaging and clinical biomarkers. With its sensitivity of 93.3% and 92.3% specificity in distinguishing between non-rejected and discharged renal transplants, the proposed method produced an accuracy of 92.9%. Using RT survivor statistics, Kyung et al. [53] conducted a retrospective study and built a predictive model to evaluate graft survival in RT receivers. Their survival decision tree model performed better compared to the conventional decision tree and Cox regression models, with indexes of concordance of 0.80, 0.71, and 0.60–0.63, respectively.

## 5. Treatment Planning

### 5.1. Prostate Cancer Radiotherapy

Brachytherapy for prostate cancer involves a systematic preparation by a brachytherapist, a time-consuming process that can have varied results, depending on the observer [54]. There has been a high degree of research involving the use of ML algorithms to rapidly build recovery schedules for brachytherapy [54,55]. The time required to create and test the algorithms was found to be much shorter (0.8 vs. 17.9 min; *p* = 0.002), while the dosimetry metrics predicted were close to that of the qualified brachytherapist [55]. The accuracy of the dosimetry may be influenced because of different geometrical complexities during external radiotherapy. AI algorithms were developed by Guidi et al. [56] to handle such issues related to avoiding radiation injuries. CT images are used to train the AI algorithms in the radiotherapy planning and recovery phase of the treatment, which are used to compare scheduled and performed radiation therapy, helping patients who thereby benefit from receiving individualized care.

### 5.2. Cancer Drug Selection

AI interventions will be of assistance in the choice of adequate medications for cancer diagnosis and treatment. Saeed et al. [57] used ML technologies to measure and assess their activity with more than 300 forms of drugs in castration-resistant prostate cancer cells. Navitoclax family inhibitor Bcl-2 was described as highly active in patients with prostate cancer resistant to castration.

### 5.3. Surgical Skill Assessment

**Figure 4:**
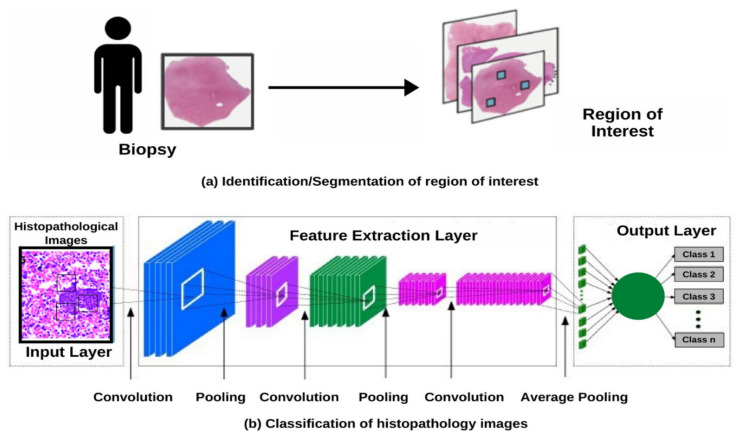
(**a**) Identification/Segmentation of the region of interest. (**b**) Classification of histopathological images using a deep learning technique.

The evaluation of medical expertise and success is usually carried out by manual peer examination, allowing professionals to evaluate the surgical success or to monitor surgical performance. Such evaluations are often unreliable and increase the uncertainty due to different definitions of success by various observers. Endoscopic instruments offer direct visualization that is integrated with video cameras. These data, along with other types of information, including the movement of the surgical instruments, can also be collected. Such imagery and output data from the surgical robot can be used to test surgical output automatically using AI techniques. Figure 4 shows the procedural representation of a general biopsy using AI techniques. Anatomical landmark identification is an important metric in the assessment of advanced surgical skills. Nosrati et al. [58] and Baghdadi et al. [59] used ML algorithms to study the color and textural features from visualization of the surgical sites’ anatomical features during partial nephrectomy and radical prostatectomy.

Tracking the movements and actions of the surgical instruments is also an important metric for performance assessment. Ghani et al. [60] looked at the movements of instruments to determine surgical skills and techniques. The authors collected data on the movements of the instruments either manually or by using motion trackers, which were then fed to an ML algorithm to determine the expertise level of the surgeon, achieving a precision between 83.3% and 100% [60].

## 6. Robotic Surgery

Apart from assessing the surgical skill, as discussed in the previous section, AI also plays a key role in improving new surgical techniques such as minimally invasive procedures involving surgical robots. Determining the best practices by analyzing the patterns and aiding in reducing technical errors are the primary tasks of AI in robotic surgery. Its performance in each sub-specialty of urology is discussed below.

### 6.1. Urologic Oncology

Recent advances in robotic urologic surgery and minimally invasive procedures have enabled approaches to treating prostate cancer, such as laparoscopic prostatectomy and robotic-assisted surgery. Robotic prostate surgery is an extremely precise procedure that provides excellent cancer control and is considered safe in experienced hands.

Radical cystectomy has been the surgical standard to treat patients suffering from muscle-invasive bladder cancer. Though there is a significant reduction in the estimated blood loss (EBL), the blood transfusion rate, and the length of stay in robotic-assisted radical cystectomy (RARC) compared to those in open radical cystectomy (ORC), the complications and the positive margin status have been found to be similar [61,62,63,64,65,66,67,68]. Although the role of RARC is controversial, it has become an acceptable alternative to open surgery by some guideline organizations, including the European Association of Urology [62].

### 6.2. Reproductive Urology

Etafy et al. [69], in a study, validated that robot-assisted microsurgical procedures are now safe and practicable in dealing with male infertility. More than 500,000 American men opt for vasectomy as a method of contraception annually, of which 2–6% will eventually undergo vasectomy reversal [70]. Studies have shown that robot-assisted vasovasostomy (RAVV) yields comparable results to that of the pure microsurgical technique [71]. Though the former approach is not superior, it offers a few additional advantages over normal surgical procedures. These benefits include the elimination of tremors, multiview magnification, additional instrument arms, and enhanced dexterity with articulating instrument arms.

### 6.3. Pediatric Urology

In pediatrics, robotic surgery remains controversial due to both cost and the lack of published high-level evidence. Ballouhey et al. [72] discussed how size difference in children cannot be a limiting factor for performing robotic surgery (patients with body weight of >15 kg or <15 kg yielded similar results). Robot-assisted laparoscopic pyeloplasty (RALP) is the standard treatment of ureteropelvic junction obstruction in older children and has even been performed in infants and redo procedures. In a study by Avery et al. [73], among the 60-patient cohort with a mean age of 7.3 months, 91% showed improvement or resolution of hydronephrosis after pyeloplasty, with 11% facing post-operative complications and 2 patients requiring redo procedures. Redo robotic pyeloplasty is deemed a safe and effective approach for recurring ureteropelvic junction obstruction, reporting up to 100% success rates and 0% complication rates [74]. Along with RALP (Robot-assisted laparoscopic pyeloplasty), robot assistance in nephrectomy [75], ureteroureterostomy [76], ureteral reimplantation [77], and other procedures has yielded affirmative results and unlocked new possibilities in the field of pediatric urology.

### 6.4. Renal Transplant

Robot-assisted RT (RART) is another application of AI that is highly recommended for obese and high-risk ESRF patients as it delivers low complication rates and excellent graft function over conventional surgery [78]. RART is considered to be a safe, feasible, and reproducible option when performed by surgeons with practice in both robotic and conventional RT surgery.

## 7. Discussion

In this article, we explored how AI can help us in the diagnosis, outcome prediction, and other treatment processes of urological diseases, even when provided with a heterogeneous and complex dataset. The growth in the granularity of data due to the huge spike in data collection over the recent years makes interpretation and pattern identification difficult for traditional statistical models, which are restricted by the limitation of using fixed correlations that work on the assumption that the data will have linear relationships. AI is much more robust and flexible when it comes to working with different data types and dealing with noise, missing data, and infrequent visits by the patient. It can even handle high-dimensionality data, while making minimum assumptions.

Although using AI can be tricky, the results and accuracy achieved when it is used correctly exceed those observed with the standard statistical models. It can also help in simplifying manually performed procedures and thus reducing the variation in outcomes due to human ability, bias, and methodological mistakes or inefficiencies. Therefore, AI-based models help clinicians in getting early, reliable, and personalized data that can help in the decision making.

It is observed that AI achieves a higher accuracy for most tasks, but it cannot be used to answer every question. Sometimes, standard statistical models can outperform AI models. Kattan et al. [79] compared ML estimation and Cox proportional risk regression methods based on three separate datasets of urological results. Cox regression could correspond with or surpass the ML model predictions. Neural networks have freely used parameters for the transformation of feature and class prediction, the neural networks being accurate and adapted to the maximal values of these free parameters. A well-constructed conventional model can outperform an ML model built lousily. Another issue with using ML-based models is something called a black box. When we make a deep neural network, the model builds non-linear, non-monotonic response functions, which despite having remarkable accuracy might be harder to explain, which makes the performance of these networks more empirical than theoretical.

Several clinicians and researchers have discussed the role of AI in healthcare and in treating certain urological conditions [80,81]. The approach adopted in this review provides a comprehensive view with an aim to address all possible aspects of AI in the field of urology. The studies reviewed by us vary in their training features, algorithms used, and the observed endpoints, which makes the task of quantitative analysis more difficult. In addition, these studies lack generalizability across different datasets as we have the results only for that particular dataset. Some of them also do not give a comparison with the standard statistical models, which limits our ability to understand how AI techniques are better than other models.

Real-life usage of AI technologies in the field of medicine is still a long way into the future. They face high levels of quality control and regulatory obstacles. The US FDA (United States Food and Drug Administration) has issued the first AI system assessment guidelines [82], which show that adaptive architecture should provide real-life evidence in clinical studies to assess the efficacy of AI techniques. AI models are data driven; they learn from the data that are given to them, and therefore require continuous training to maximize their utility and accuracy.

## 8. Conclusions

AI has come a long way in making exponential progress in healthcare over the past decade. There are still a lot of challenges and hurdles that need to be addressed before these techniques can be completely trusted to be used in the medical field. Though the future of AI in the field of urology is bright, considering it has already provided excellent solutions to handle various health issues through early diagnosis and personalized treatment, there is still a lot of room for improvement and growth when it comes to delivering solid results to positively influence more number of lives on an individualized basis.

## Figures and Tables

**Table 1:** Studies using AI to diagnose prostate cancer.

Study	Application of the Study	Type of Study	Size of the Sample Used	Features Used for Training	Algorithms Used	Accuracy, %	Sensitivity, %	Specificity, %	AUC
Kim et al., 2017 [6]	Forecast of extracapsular expansion	Retrospective	944 patients (621 and 323 organ-confined disease and non-organ-confined disease, respectively)	PSA, Gleason score, clinical T stage, and positive prostate biopsy core count	NN	73.4	-	-	-
					SVM	75.0	-	-	-
					NB	74.8	-	-	-
					BNs	74.4	-	-	-
					CART	70.7	-	-	-
					RF	68.8	-	-	-
Algohary et al., 2018 [7]	Diagnosis based on MRI	Retrospective	56 patients	Radiomic MRI features chosen by unsupervised hierarchical clustering	QDA	72.0	75.0	60.0	-
					RF	32.0	42.0	30.0	-
					SVM	52.0	60.0	40.0	-
Ginsburg et al., 2017 [8]	Diagnosis based on MRI	Retrospective	80 patients	Radiomic MRI characteristics	LR	-	-	-	0.61–0.71
Fehr et al., 2015 [10]	Forecast of Gleason score using MRI	Retrospective	356 regions of interest from 147 patients	Radiomic MRI characteristics	*t*-Test SVM (Gleason 6 vs. ≥7)	73–83	-	-	0.83–0.90
					AdaBoost (Gleason 6 vs. ≥7)	64–73	-	-	0.60–0.74
					RFE-SVM (Gleason 6 vs. ≥7)	83–93	-	-	0.91–0.99
					*t*-Test SVM (Gleason 3 + 4 vs. 4 + 3)	66–81	-	-	0.94–0.99
					AdaBoost (Gleason 3 + 4 vs. 4 + 3)	73–79	-	-	0.75–0.80
					RFE–SVM (Gleason 3 + 4 vs. 4 + 3)	83–92			0.77–0.81
Kwak et al., 2017 [11]	Diagnosis based on images of tissue samples	Retrospective	653 tissue samples	HE-stained digitized images of the prostate specimen	Multiview boosting classifier (differentiate benign and malignant tissue)	-	-	-	0.98
					Multiview boosting classifier (differentiate epithelium and stroma)	-	-	-	0.97–0.99
Kwak et al., 2017 [12]	Diagnosis based on images of tissue samples	Retrospective	827 tissue samples	HE-stained digitized images of the prostate specimen	CNN	-	-	-	0.97
Nguyen et al., 2017 [13]	Estimation of Gleason score based on tissue samples from the prostate	Retrospective	368 prostate tissue samples (1 per patient)	HE-stained digitized images of the prostate specimen	RF (benign vs. malignant)	-	-	-	0.97 0.82
					LR (Gleason scoring 3 vs. 4)	-	-	-	0.82

Area Under the ROC Curve (AUC); Neural Network (NN); Support Vector Machine (SVM); Prostate Specific Antigen (PSA); Naive Bayes (NB); Bayesian Networks (BNs); Classification and Regression Tree (CART); Random Forest (RF); Quadratic Discriminant Analysis (QDA); Magnetic resonance imaging (MRI); Logistic Regression (LR); Recursive Feature Elimination (RFE); Hematoxylin and Eosin (HE).

**Table 2:** Studies using AI to diagnose urothelial cancer.

Study	Application of the Study	Type of Study	Size of the Sample Used	Features Used for Training	Algorithms Used	Accuracy, %	Sensitivity, %	Specificity, %	AUC
Xu et al., 2017 [14]	Differentiate bladder tumor and bladder wall tissue by MRI	Retrospective	62 patients (62 cancerous regions and 62 bladder wall regions)	Radiomic MRI characteristics: 2D texture characteristics and 3D texture characteristics	SVM (2D)	70.16–78.23	-	-	0.72–0.83
					SVM (3D)	71.77–85.48	-	-	0.77–0.89
					RF (2D)	70.16–79.84	-	-	0.72–0.82
					RF (3D)	68.56–85.48	-	-	0.73–0.87
					SVM (RFE-selected optimal features)	87.9	90.3	85.5	0.90
Garapati et al., 2017 [15]	Forecast the stage of the disease based on CT urography	Retrospective	76 CT urography cases (84 bladder cancer lesions: 43 < T2; 41 ≥ T2)	Pathological stage, CT urography morphological features, and textural features	LDA (training set)	-	-	-	0.91
					LDA (testing set)				0.88
					SVM (training set)				0.91
					SVM (testing set)				0.89
					RF (training set)				0.89
					RF (testing set)				0.97
					NN (training set				0.89
					NN (testing set)				0.92
Shao et al., 2017 [16]	Forecast whether the disease is present or not	Prospective	87 bladder cancer patients and 65 patients without bladder cancer	6 urine metabolite markers (spectral ions)	DT: testing	76.6	71.9	86.7	-
					DT: training (5-fold cross validation)	84.8	81.8	88.0	-
Ikeda et al., 2019 [17]	Detect tumors	Retrospective	422 cystoscopic images	Transfer learning using features extracted from gastroscopic images	CNN	-	96.5	96.5	-

Computed Tomography (*CT*); convolutional neural network (CNN).

**Table 3:** Studies using AI to diagnose renal cancer.

Study	Application of the Study	Type of Study	Size of the Sample Used	Features Used for Training	Algorithms Used	Accuracy, %	Sensitivity, %	Specificity, %	AUC
Zheng et al., 2016 [18]	Forecast the presence of the disease in the earlier stages	Retrospective	126 patients (68 healthy participants and 48 renal cell cancer (RCC) patients)	Serum metabolome biomarker cluster	ANN: healthy participants	91.3	-	-	-
					ANN: RCC	94.7	-	-	-
Haifler et al., 2018 [19]	Discriminate between normal and malignant renal tissue	Prospective	6 clear-cell RCC specimens; 6 normal kidney tissue specimens	Short-wave infrared Raman spectroscopy	SMLR	92.5	95.8	88.8	0.94

Sparse Multinomial Logistic Regression (SMLR).

**Table 4:** Studies using AI to predict outcomes of prostate cancer.

Study	Application of the Study	Type of Study	Size of the Sample Used	Features Used for Training	Algorithms Used	Accuracy, %	Sensitivity, %	Specificity, %	C-index	AUC
Lam et al., 2014 [43]	Forecast mortality for a period of 5 years after radical cystectomy	Retrospective	117 patients (83 training, 17 validation, and 117 testing)	Age, tumor stage, albumin level, surgical approach	ANN	77.8	-	-	-	0.829
Wang et al., 2015 [44]	Forecast mortality for a period of 5 years after radical cystectomy	Retrospective	117 patients	Gender, age, age range, albumin, surgical approach 1/2, preoperative albumin, tumor stage, follow-up period, type of diversion	NN	72.2	77.6	68.1	-	-
					ELM	76.7	73.5	81.5	-	-
					RELM	80.0	85.6	72.4	-	-
					RBF	76.7	79.0	75.3	-	-
					SVM	75.6	75.4	77.0	-	-
					NB	73.3	73.8	73.4	-	-
					k-NN	72.2	75.1	70.1	-	-
Sapre et al., 2016 [45]	Predict urothelial carcinoma recurrence	Prospective	Training set 81 patients (21 benign controls, 30 no recurrence, and 30 active cancer recurrence); testing set 50 patients	Urinary miRNAs (miR205, miR34a, miR21, miR221, miR16, miR200c)	SVM (recurrence)	-	88.0	48.0	-	-
					SVM (tumor presence): training	-	-	-	-	0.85
					SVM (tumor presence): testing	-	-	-	-	0.74
					SVM (T1)	-	-	-	-	0.92
					SVM (Ta)	-	-	-	-	0.72
					SVM (T2,3,4)	-	-	-	-	0.73
					SVM (high volume)	-	-	-	-	0.81
					SVM (low volume)	-	-	-	-	0.69
					SVM (low grade)	-	-	-	-	0.76
					SVM (high grade)	-	-	-	-	0.75
					SVM (initial tumor)	-	-	-	-	0.76
Bartsch et al., 2016 [46]	Estimate the risk of recurrence in 5 years for non-muscle-invasive urothelial carcinoma after transurethral resection of the bladder	Retrospective	112 frozen non-muscle-invasive urothelial carcinoma specimens	Genes in DNA sampling	GP (3-gene rule): training	-	80.4	90.0	-	-
					GP (3-gene rule): testing	-	70.6	66.7	-	-
					GP (5-gene combined rule): training	-	77.1	84.6	-	-
					GP (5-gene combined rule): testing	-	68.6	61.5	-	-

Regularized Extreme Learning Machine (RELM); MicroRNA (miRNA); Glycoprotein (GP).

**Table 5:** Studies using AI to predict outcomes of urothelial cancer.

Study	Application of the Study	Type of Study	Size of the Sample Used	Features Used for Training	Algorithms Used	Accuracy, %	Sensitivity, %	Specificity, %	C-index	AUC
Wong et al., 2019 [35]	Estimate the recurrence of the disease after radical prostatectomy	Prospective	338 patients	Patient clinicopathology information	k-NN	97.6	78.0	69.0	-	0.903
					RF	95.3	76.0	64.0	-	0.924
					LR	97.6	75.0	69.0	-	0.94
Harder et al., 2018 [36]	Estimate the recurrence of the disease after radical prostatectomy	Retrospective	90 patients (40 with PSA recurrence)	Tissue phenomics of the disease	Hierarchical clustering	86.6	82.5	90.0	-	-
					naive Bayes	83.3	80.0	86.0	-	-
					classification and regression tree	83.3	70.0	94.0	-	-
					k-NN	85.5	80.0	90.0	-	-
					Linear predictor	87.8	94.0	80.0	-	-
					SVM (linear kernel)	86.7	77.5	94.0	-	-
					SVM (radial bias function kernel)	82.0	75	88.0	-	-
Zhang et al., 2016 [37]	Estimate the recurrence of the disease after radical prostatectomy	Retrospective	205 patients (61 with biochemical recurrence)	Radiomic MRI characteristics	SVM	92.2	93.3	91.7	- - - - -	0.96
Shiradker et al., 2018 [38]	Predict the biochemical recurrence of prostate cancer using MRI	Retrospective	120 patients (70 training; 50 validation)	Patient clinicopathological data and radiomic MRI characteristics	LDA (radiomic alone, training)	-	-	-	0.54	-
					SVM (radiomic alone, training)	-	-	-	0.84	
					RF (radiomic alone, training)	-	-	-	0.52	
					SVM (radiomic alone testing)	-	-	-	0.73	
					SVM (radiomic + clinical training)	-	-	-	0.91	
					SVM (radiomic + clinical testing)	-	-	-	0.74	
Zhang et al. 2017 [39]	, Estimate biological recurrence after radical prostatectomy	Retrospective	424 patients (58 with recurrence)	Somatic gene mutation profiles	SVM (genetic signature alone)	66.2	-	-	-	0.7
					SVM (genetic signature + clinicopathological features)	71.3	-	-	-	0.75
Lalonde et al. 2014 [40]	Predict the biochemical recurrence after radiation or radical prostatectomy	Retrospective	397 patients (126 training, 154 validation, and 117 testing)	Genes of the disease, general genomic instability, and tumor microenvironment	RF (validation set 1)	-	-	-	0.7	0.74
					RF (validation set 1)	-	-	-	0.74	0.84
					RF (validation set 2)	-	-	-	0.67	0.64
					RF (validation set 2)	-	-	-	0.73	0.75
Hung et al. 2018 [41]	Predict the length of stay required in the hospital after radical prostatectomy	Ambispective	78 patients	25 surgical robotic APMs	RF	87.2	-	-	-	-
					RF (APMs and patient demographics)	88.5	-	-	-	-
					SVM	83.3	-	-	-	-
					LR	82.1	-	-	-	-
Hung et al. 2018 [42]	Predict urinary continence recovery after robotic radical prostatectomy	Ambispective	79 patients	16 clinicopathological features and 492 robotic APMs	Random survival forests, Deep-learning-model-based survival analysis	-	-	-	0.58	-
						-	-	-	0.6	-

Automated Performance Metrics (APM).

## Data Availability

Not applicable.
